# Langerhans Cell Histiocytosis Causing Central Nervous System Vasculitis Leading to Stroke in a Two-year-old Boy: A Case Report

**DOI:** 10.7759/cureus.3951

**Published:** 2019-01-24

**Authors:** Nathan T Froelich, Elias Rizk

**Affiliations:** 1 Neurosurgery, Penn State Hershey Medical Center, Hershey, USA

**Keywords:** langerhans-cell histiocytosis, cns vasculitis, stroke

## Abstract

Here, the authors present the first documented case of a patient developing central nervous system (CNS) vasculitis secondary to Langerhans cell histiocytosis (LCH) ultimately leading to stroke. LCH is a rare histiocytic disorder affecting males and females equally and typically presents in pediatric patients with a median age of 30 months. Presentation of the disease can be single-site or multisystem; and, classification of treatment is further demarcated by high risk and low risk depending on the organ systems involved. Treatment of LCH typically involves vinblastine and prednisone, as well as salvage treatment as needed.

## Introduction

Langerhans cell histiocytosis (LCH) is a rare histiocytic disorder of unknown etiology affecting 2-10 per one million children younger than age 15 years, with a median age of presentation of 30 months and a roughly equal male: female ratio [1-2]. LCH typically presents with a skin rash and/or bone lesion and can affect single or multiple organ systems including marrow, liver, and lungs. Common presentations of a lytic bone lesion secondary to LCH include cervical lymphadenopathy (40.9%) and otitis media (22.7%) [3]. It can also affect the lungs and the central nervous system (CNS). Diagnosis of LCH is confirmed with positive staining of biopsy sample with immunohistochemistry staining of CD1a, S100, and CD207 (Langerin). BRAFV600E mutations are common in pediatric LCH patients as well [4-5]. Treatment of LCH is typically a combination of prednisone and vinblastine. In addition to treatment side effects, other morbidities depend on whether the disease affects a single system (24% incidence of long-term problems) or multiple systems (71% incidence of long-term problems) [6]. While other histiocytic diseases have been known to cause CNS vasculitis and stroke [7-8], this is the first documented case of LCH presenting with an acute ischemic stroke.

## Case presentation

**Figure 1:**
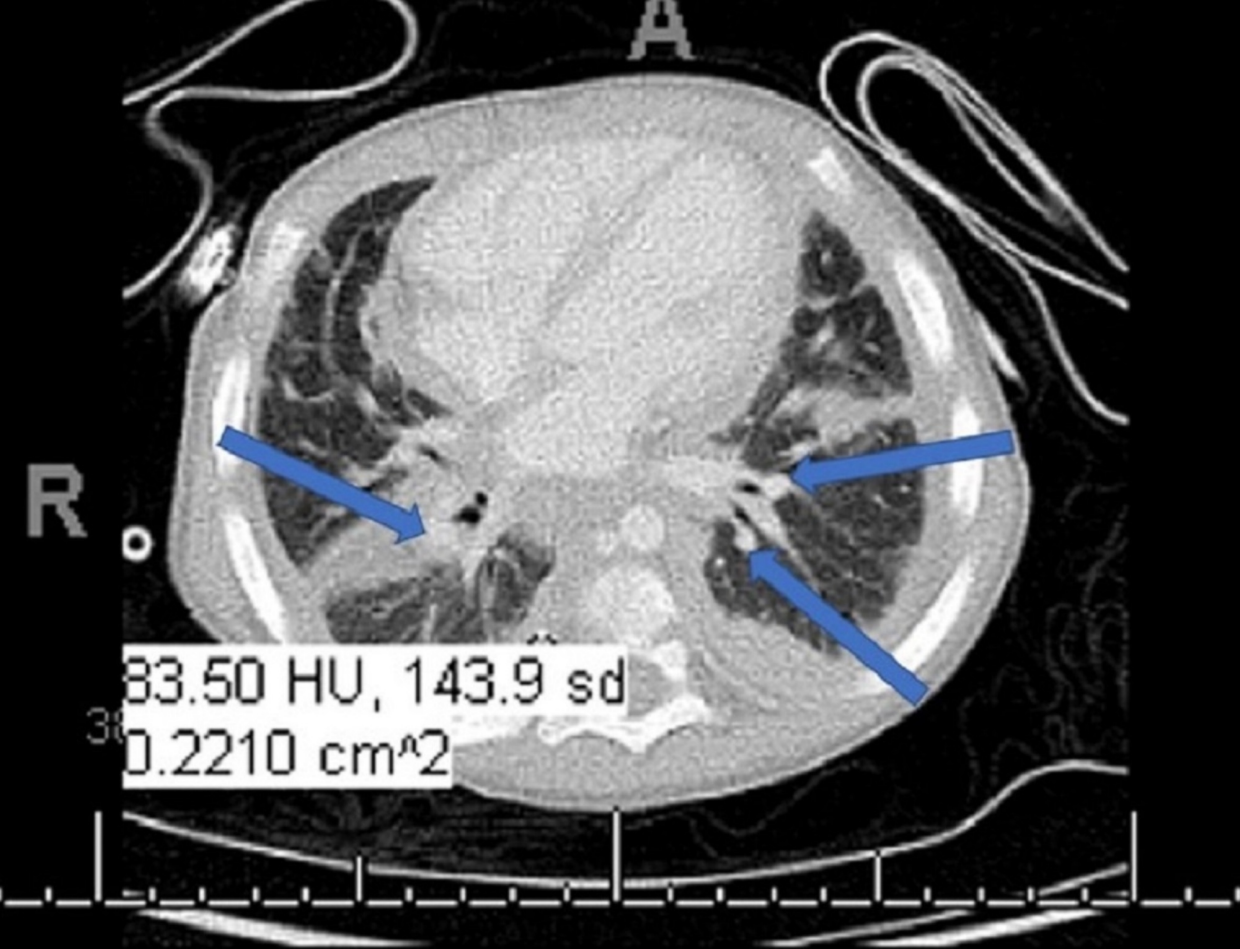
Computed tomography (CT) showing cardiomegaly with diffuse interstital thickening suggestive of pulmonary interstitial edema. Cardiomegaly with diffuse interstitial thickening suggestive of pulmonary interstitial edema with small bilateral pleural effusions. Findings suggest underlying cardiac pathology; groundglass opacities in the right middle and lower lobes are concerning for infectious versus inflammatory process; scattered subcentimeter mediastinal lymph nodes, the most conspicuous subcarinal node measuring 0.9 cm. Differential favors reactive process; Atelectasis adjacent to the periphery of the lung and fissures.

A 16-month-old male presented to the emergency department (ED) with tachypnea and dyspnea. Left-sided lymphadenopathy was found on examination, as well as a large mass was found on the lateral frontal bone just above the orbit. The patient’s mother noted that it had been growing for six weeks; and, ultrasound of the mass showed it was cystic in nature. On history, the patient had seven to eight documented ear infections within the previous year. Chest X-ray (CXR) found indications of heart failure (HF) (enlarged heart and interstitial edema typical of HF); however, echocardiogram demonstrated normal heart structure and function. Computed tomography (CT) showed hilar and subcarinal lymphadenopathy (Figure 1).

**Figure 2:**
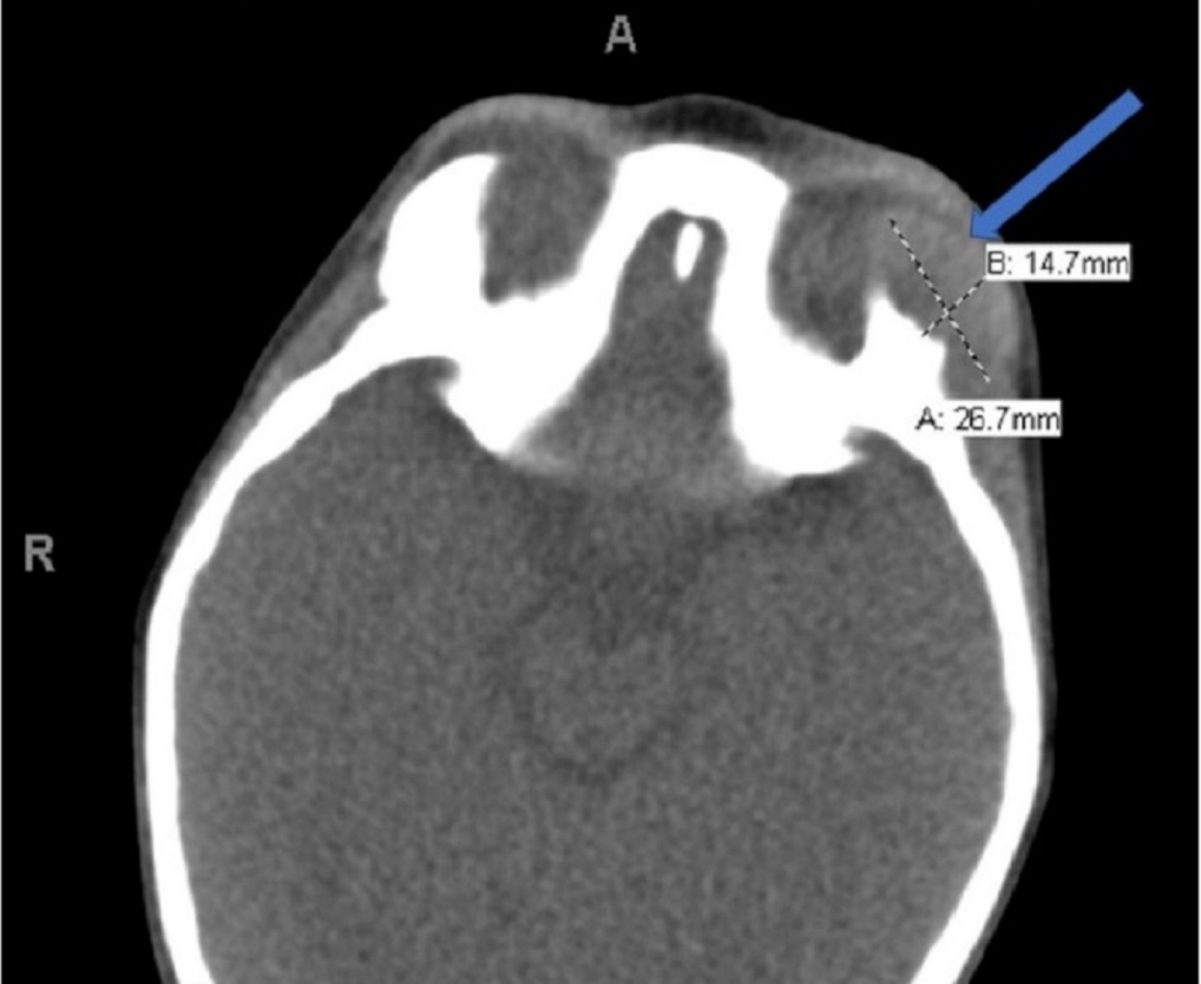
CT of lytic lesion concerning for eosinophilic granuloma (EG) or hematologic malignancy (HM). Sharply defined lytic lesion zygomatic process of the left frontal bone forming the superolateral wall of the left orbit with intraorbital extension; also extension to the left temporal fossa as described. Findings probably indicate eosinophilic granuloma. Differential is hematologic malignancy; mild left proptosis; hematologic workup requested for further assessment; biopsy of the lesion with imaging guidance can be considered following metastatic and hematologic workup.

The patient was treated with ceftriaxone and zithromax. After treatment showed limited effectiveness (persistent infection and fever of 103°F), the patient underwent bronchoalveolar lavage (BAL). BAL indicated airways with no active infection or malignant cells; however, there were signs of inflammation. Head CT showed sharply defined lytic lesion of the left orbit region, concerning for eosinophilic granuloma or hematologic malignancy (Figure 2).

Biopsy was performed; immunohistochemistry was positive for CD1a, S100, CD68, and CD207 (Langerin). The patient was also positive for the BRAFV600E mutation. With this information a diagnosis LCH was confirmed and therapy was initiated with vinblastine and prednisone. After his first dose of chemotherapeutic treatment, he was discharged from the hospital.

**Figure 3:**
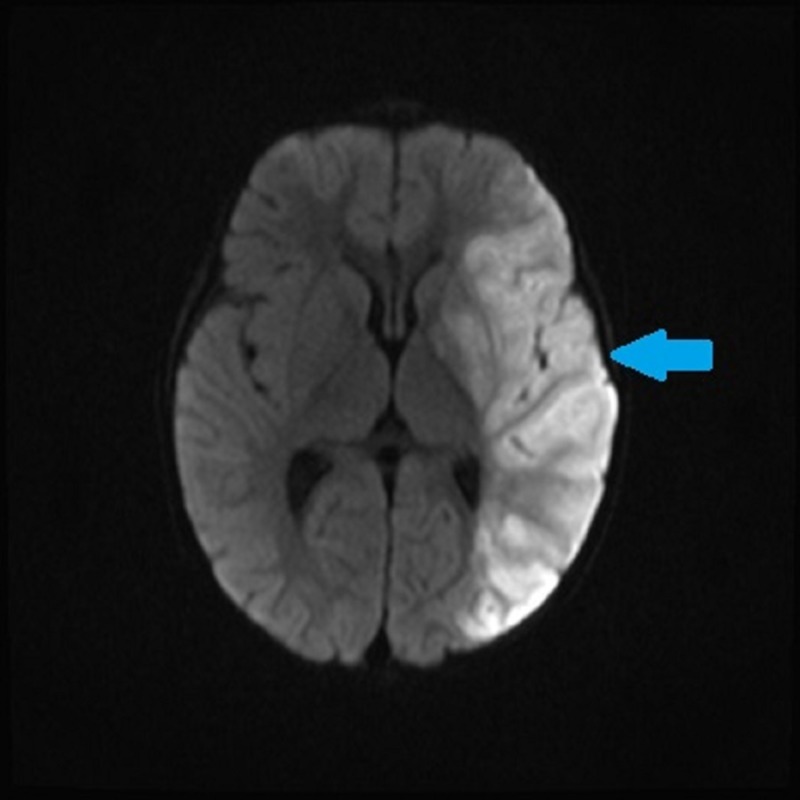
Magnetic resonance angiogram (MRA) with contrast showing left middle cerebral artery (MCA) territory infarction. Large left MCA territory infarction with acute cutoff of left MCA; significant decrease in the caliber of the left internal carotid artery throughout its intracranial course.

**Figure 4:**
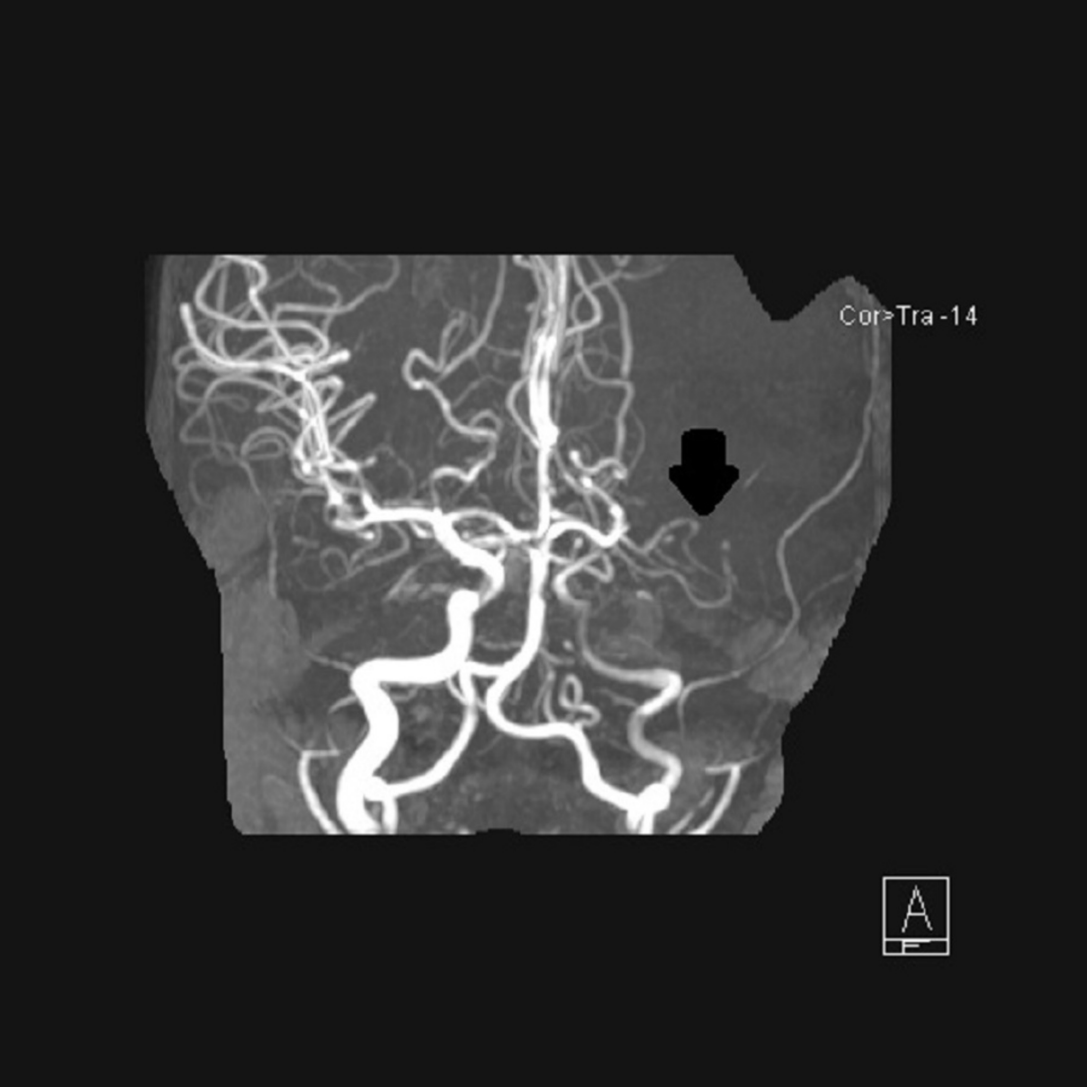
MRA showing occlusion of MCA. Interval evolution of large left MCA distribution full-thickness infarction with mild increase in size and mass effect. No midline shift; persistent occlusion of left MCA and diffuse narrowing of the cervical, petrous, cavernous, and supraclinoid internal carotid artery (ICA) likely secondary to vasculitis. Additional foci of multifocal narrowing in the anterior and posterior circulation also indicated of vasculitis; no focal intramural hematoma to suspect dissection; no intraluminal thrombosis in the neck to suspect thrombosis or embolism from the cervical ICA; interval marked decrease in periorbital soft tissue mass histiocytosis by pathology.

Approximately one week after initiating chemotherapy, the patient was re-admitted to the hospital with left brain stroke. Head and neck magnetic resonance imaging/magnetic resonance angiogram (MRI/MRA) found large left middle cerebral artery (MCA) territory infarction, with acute cutoff of left MCA and significant decrease in caliber of the left internal carotid artery (ICA) throughout its intracranial course (Figures 3-4).

Echocardiogram demonstrated a functionally and structurally normal heart with intact atrial septum; and, no thrombus or vegetation was noted. Following an extensive workup and ruling out all other possible causative factors, the etiology of stroke was attributed to secondary CNS vasculitis. This was possibly secondary to his primary diagnosis of LCH.

## Discussion

Though uncommon in the pediatric population, there are reports in adults of histiocytic disorders leading to CNS vasculitis (Table 1) [9-10]. Vasculitides in children is a rare condition as a whole, occurring in no more than 53 in 100,000 (12401245, 15517648). LCH is even rarer still [TF1] with reports of nine cases per million in this age group (16652350).

Therefore, it is extremely unlikely that these two medical conditions occurred independently of each other. Finally, vasculitis secondary to chemotherapy has been documented with gemcitabine but not with vinblastine [11]. Therefore, the likelihood that vinblastine contributed to the stroke is unlikely. Hence this would be the first case describing vasculitis in the setting of LCH and a subsequent stroke in a 16-month-old child. 

**Table 1:** Summary of relevant vasculitides affecting central nervous system (CNS). *Abbreviations*: UE, upper extremity; CSF, cerebrospinal fluid; MRI, magnetic resonance imaging.

CNS Vasculitis	Pathophysiology	Clinical presentation	Long-term consequences
Takayasu	Large-vessel vasculitis affecting branches of the aortic arch	Depending on the site of inflammation; includes decreased UE blood pressure, brain ischemia	Ischemia to UE or branches of the carotid arteries
NP-cPACNS (16575852)	Exact etiology unknown, thought to be inflammatory in nature	Sudden-onset, nonfocal neuro deficits, 40% incidence of headaches, inflammatory markers, CSF usually normal. MRI show l lesions in large vessels.	Focal neurologic deficits
P-cPACNS (16575852)	Exact etiology unknown, thought to be inflammatory in nature	Focal and diffuse neuro deficits, often affecting more than one region (16418382), 95% incidence of headaches, inflammatory markers, CSF sometimes abnormal.	Focal neurologic deficits, global deficits (e.g. personality changes)
SV-cPACNS (16575852)	Exact etiology unknown, thought to be inflammatory in nature	Severe encephalopathy, extensive focal deficits, all seizure types with severity including status epilepticus. inflammatory markers, CSF usually abnormal.	Focal neurologic deficits, global deficits (e.g. personality changes)

## Conclusions

This would be the first case in pediatrics or adults describing vasculitis in the setting of LCH and a subsequent stroke. Clinicians should be aware that this devastating outcome can be a rare presentation in the setting of LCH.
